# Perception of men's need for preconception care—A qualitative exploration among health care providers and community members

**DOI:** 10.3389/fpubh.2022.958618

**Published:** 2022-11-29

**Authors:** Oludoyinmola O. Ojifinni, Latifat Ibisomi

**Affiliations:** ^1^School of Clinical Medicine, Faculty of Health Sciences, University of the Witwatersrand, Johannesburg, South Africa; ^2^Division of Epidemiology and Biostatistics, School of Public Health, University of the Witwatersrand, Johannesburg, South Africa; ^3^Monitoring and Evaluation Department, Nigerian Institute of Medical Research, Lagos, Nigeria

**Keywords:** preconception care, men's reproductive health, preconception care need, Nigeria, men's attitude to health care

## Abstract

**Background:**

Several studies have shown that suboptimal health in men can result in poor reproductive health outcomes. The factors associated include lifestyle exposures and poor health-seeking behavior. The poor reproductive health outcomes can be mitigated through preconception care (PCC). PCC services for men are however rare. This qualitative study explored views about men's need for PCC in Nigeria.

**Methods:**

This exploratory qualitative study was done in Ibadan North Local Government Area, Oyo State, Nigeria. Focus group discussions were held with 12 religious leaders, 22 men and 23 women of reproductive age at the community level. There were key informant interviews with two community leaders and 26 health workers including specialist physicians and nurses at the primary, secondary, and tertiary health care levels. Transcribed data were analyzed thematically using inductive coding on MAXQDA.

**Results:**

The reasons participants proffered for men's health requiring attention included men's genetic contribution to pregnancy, treatment of low sperm count, and preventing transmission of infection to their partners. Participants stated however that men are often reluctant about accessing health services until complications arise. Opinions differed on men's need for PCC: while some believed that men need PCC, others expressed contrary views stating that men do not require PCC as the service is more appropriate for women.

**Conclusion:**

Successful deployment and uptake of PCC services require the availability of the services and improved awareness about the need to optimize men's health along with that of their partners.

## Introduction

The preconception period refers to the time when a couple of reproductive age has the potential to achieve conception (1). The concept of preconception health refers to an individual's biopsychosocial wellness during the preconception period (1, 2). Preconception care (PCC) is the provision of biomedical, behavioral, and social health interventions to women and couples before conception occurs, aimed at improving their health status, and reducing behaviors and individual and environmental factors that could contribute to poor maternal and child health outcomes (3, 4). Although women have been the target of PCC programs, the importance of PCC for men is gaining momentum because their preconception health affects their biological, and genetic contributions to pregnancy outcomes (5–7). PCC interventions for men include a reproductive health plan, health assessment, health promotion, and clinical and psychosocial interventions to improve adolescent and young men's health (5, 8–10). PCC for men serves a dual function—it optimizes men's overall health through disease prevention and health promotion interventions and encourages them to support women's preconception health status and reproductive plans (5, 10–13). Men also need reproductive planning with their partners, a component of PCC that assists individuals and couples to decide if, when, and how many children they want to have while ensuring health is optimized before conception (14, 15). Additionally, involving men in reproductive life planning through preconception counseling can ensure better preparation for parental responsibilities and improve reproductive health outcomes by encouraging positive perinatal care choices and health-seeking behavior while supporting maternal health decisions (7). More importantly, PCC for men is an opportunity to improve men's overall health through health promotion and disease prevention interventions (5).

Male involvement in reproductive health issues has however been hampered by men's view that women have more rights in reproductive health since they are the ones who get pregnant (16, 17). Studies among men in the United Kingdom, the United States, and Netherlands reported that men believed they do not have a voice in reproductive health issues, and as such discussions typically focus on women (16–18). A systematic review of studies on men's knowledge and attitudes toward fertility issues revealed that men generally have poor knowledge about fertility and the factors that can influence conception (19). Several studies have however shown that environmental and lifestyle exposures in men are associated with changes in the sperm genome (7, 10, 20–22). These changes have been linked with low sperm motility, increased time to pregnancy in partners, the subsequent occurrence of birth defects, and a negative impact on the future health of children (7, 10, 22, 23). The implicated environmental exposures and lifestyle issues can be addressed during the preconception period, a critical window when both paternal and maternal health can be optimized to improve reproductive health outcomes (24, 25).

Beyond optimizing men's health, reproductive planning is an important aspect of PCC in paternalistic societies like Nigeria where men often desire more children than women. The 2018 Nigeria Demographic and Health Survey (NDHS) reported that men desired at least one child more on average compared with women (26). PCC through reproductive planning can open up conversations around fertility preferences and address the discrepancy in the desire for more children among couples (27). This is particularly critical given that parenthood is culturally believed to signify maturity in many Nigerian communities and is only socially acceptable within marriage (28–30). Within the community setting in Nigeria therefore, and among the Christian and Islamic religious bodies, anecdotal reports state that leaders often provide premarital counseling to couples who are preparing for marriage. Some of the content of the counseling has been reported to include aspects of preconception care (30).

PCC services are however not yet established in many low- and middle-income countries (LMICs) including Nigeria. Existing PCC studies have assessed the provision of PCC by health workers and uptake among women of reproductive age. Evidence shows that where available, PCC services are provided opportunistically or as requested by clients, who are mostly women (30, 31). There is still a gap in the understanding of PCC services for men. With the increasing awareness of the vital role men play in reproductive health, it is critical to include men in conversations about PCC while the services are in the initial stages of development and deployment. This study, therefore, explored the perception about men's need for PCC among adult men and women, religious and community leaders, and health care providers in Nigeria. The data for this article is part of a study that explored the need for and feasibility of PCC services within the Nigerian health system.

## Materials and methods

This exploratory qualitative study followed an ontological assumption that each potential participant has a different understanding of men's need for PCC based on their personal experiences (32). The exploration, therefore, shows multiple perspectives about men's need for PCC in Nigeria. The views of different individuals within the health system and at the community level are presented as themes developed from the data (32). The study employed focus group discussions (FGDs) and key informant interviews (KIIs). The findings are triangulated with emphasis placed on the practical implications of these varying perspectives (32–34).

### Study area

Politically, Nigeria is divided into 36 states and the federal capital territory. Oyo State where the study was done is located in the southwest and has 33 local government areas (LGAs), five of which are within Ibadan, the capital city. The study was conducted in Ibadan North LGA, one of the urban LGAs selected because it has health facilities at the primary, secondary, and tertiary levels of care. The Nigerian health system operates at three levels of specialization increasing from primary to secondary and tertiary. The services provided in the Nigerian health care system include basic health services including primary prevention and treatment of minor ailments at the primary level. The primary health centers are located at the ward level of political administration and are supervised by Ward Development Committees as part of the community participation arm of primary health care (35, 36). Specialized care is offered at the secondary and tertiary levels of health care (37, 38). There is a two-way referral system through which patients are referred to higher levels when more specialized services are needed or stepped down to lower levels when the specialized services are no longer required (35, 36). The roles played by the health workers at the three levels, therefore, increase in complexity depending on whether they are at the primary, secondary or tertiary level of health care.

### Study population and participant selection

The study population included health workers at the primary, secondary, and tertiary levels of care as well as community members including community and religious leaders. Details of the participant distribution are shown in Table 1. The health workers were purposively selected based on their specialties and roles in providing health services to men and women in the reproductive age bracket. Recruitment of health care workers at the three levels of care was to ensure adequate representation of the varying roles within the health system. The specialties represented in the study sample are shown in Table 1. The number of participants at the three levels reflects the distribution of health workers within the health system in the selected LGA. Inclusion of community and religious leaders was because of their involvement in providing premarital counseling within their communities and religious organizations. The community leaders recruited for this study were identified from the Ward Development Committee overseeing the primary health care center within the study area. The religious leaders were recruited through the local branch of the national umbrella Christian and Muslim organizations. The community leaders assisted with the recruitment of the community members purposively selecting men and women between 18 and 49 years.

**Table 1:** Distribution of the study population.

**Groups**	**Sub-groups**		**No**
Community level	Adult women		23
	Adult men		22
Community leaders	Woman		1
	Man		1
Religious leaders	Christian		7
	Muslim		5
Health workers	Primary	Medical Officer of Health	1
		Assistant director for maternal and child health services	1
		Clinical nurse/midwife in the LGA primary health center	1
	Secondary	Gynecologist/Obstetrician	2
		Pediatrician	1
		Clinical nurse/midwife	2
	Tertiary	Community Medicine	2
		Gynecology and obstetrics	2
		Pediatrics	2
		Cardiology	1
		Nephrology	1
		Neurology	1
		Endocrinology	1
		Psychiatry	1
		Hematology	1
		Public Health nurse	3
		Clinical nurse	3
Total			**85**

### Data collection

Using information obtained from PCC literature, interview guides with open-ended questions were prepared for the study. The guides for the community-level interviews were translated into Yoruba, the local language and back-translated to ensure consistency of meaning. Two qualitative researchers participated in the content validation of the tools, after which the guides were pretested, and ambiguous questions deleted or modified before data collection. The participants selected their preferred language for the interviews/discussions and the version of the tool used (English or Yoruba) depended on their selection. As an introductory question, the health workers were asked “In your opinion, do women of childbearing age require care that is different in any way from other patients who are seen in your practice? How about men between ages 15 and 59 years?” For the FGD participants at the community level, the introductory question was “What are the things that you believe are important for the health of couples who are trying to have a baby?” The participants were then asked to provide their opinion about what PCC involves—definitions and components. These findings have been published elsewhere (30).

For clarity and consistency across the interviews and for the diverse study participants, the following explanation of PCC was provided: Preconception care is a special type of care provided for women of reproductive age before pregnancy to detect, treat or counsel them about pre-existing medical and social conditions that can endanger pregnancy. Preconception care for men is also important as their health affects their biologic and genetic contributions to pregnancy outcomes. The goal of preconception care is to ensure that the parents are in an optimal state of health before pregnancy occurs. It includes screening, counseling, and treatment of pre-existing medical conditions as well as reproductive life planning. Following this description, the participants were asked to provide their opinion about the relevance of PCC services to men's health. The probes applied in reference to the explanation of PCC provided included: “To what extent would you say an understanding of preconception care is important to men's health? In your opinion, how much do men require distinct preconception care services?”

The first author supervised the data collection. Having worked with many of the health care providers previously, she did not participate directly in the data collection to avoid influencing the participants' responses. A team of research assistants including Masters Students and recent Masters Graduates in the Faculty of Public Health of the University of Ibadan, Nigeria facilitated the face-to-face interviews between March and October 2018. To ensure consistency in the data collection, the same set of facilitators conducted the IDIs and FGDs. The facilitators included three women and three men working in pairs who had prior experience in conducting qualitative interviews but no prior experience in PCC services. A 1-day training aimed specifically at familiarizing the research assistants with the concept of PCC and the data collection tools was held before data collection. The male facilitators led the interviews and FGDs with the male participants while the female facilitators led the interviews and FGDs with the female participants. The facilitators made field notes which were discussed during the debriefing meetings held with the first author for feedback, review, and planning of subsequent interviews. None of the research assistants had any prior relationship with the study participants. The interviews were conducted at a location of preference for the participants, a venue within the community for the FGDs, and the offices of the health workers. The discussions and interviews lasted between 30 min to an hour. There were eight focus group discussions (FGDs) with the community members who were grouped by sex (male and female), marital status (single and married), and educational status (basic education and above and less than basic education). This categorization of participants at the community level was to ensure some homogeneity and encourage freedom of expression during the discussions (39, 40). Two FGDs were held with the religious leaders (Christian and Muslim), two in-depth interviews (IDIs) with the community leaders (male and female), and 26 IDIs with the health care providers. Data collection was discontinued when new information was no longer being obtained from the interviews implying that saturation had been achieved (41).

### Data management and analysis

The facilitators transcribed the interview recordings verbatim and translated as needed (for those conducted in Yoruba). The recordings were saved in a password-protected computer accessible only to the authors and deleted off the digital recorders. The first author read the transcripts, integrated them with the facilitators' field notes, removed transcription errors from the data and edited where necessary to ensure consistency with the audio recordings. The health worker transcripts were returned to the participants for review and the minimal (mainly editorial), corrections made were effected after which the transcripts were de-identified. This member-check was to improve the credibility of the data by ensuring accuracy of the transcripts prior to coding and analysis (42). For the FGDs, the facilitators summarized responses as the discussions progressed to ensure the viewpoint expressed was understood and correctly captured. Thematic analysis using inductive coding was applied in analyzing the data (43, 44). To avoid bias and enhance trustworthiness in the analysis, two independent coders who are not authors on this paper read through a sample of the transcripts and developed codes inductively. A consensus meeting between the independent coders and the first author was held to discuss the codes and reach an agreement on code definitions and themes. The total of 15 codes identified were merged into four main themes and subthemes which the first author applied to the data and supportive quotes were identified for inclusion in the paper. The data analysis was done using MAXQDA 2018 (45). The preparation of this article was guided by the Consolidated Criteria for Reporting Qualitative Research (COREQ) (41).

### Ethical considerations

The study was performed following the principles of the Declaration of Helsinki. Participants were provided with an information sheet detailing the study process and implications. Participation was voluntary with minimal risks; consent both for participation and recording of the interviews or discussions was obtained from all participants. No identifying data was collected, and the transcripts were saved in a password-protected computer accessible only to the authors. Ethical approval was received from the Oyo State Ministry of Health (Approval number AD/13/479/565), the University of Ibadan/University College Hospital (UI/UCH) Institution Review Board (UI/EC/17/0390), and the Wits Human Research Ethics Committee (Medical; Clearance number M171054).

## Results

### Sociodemographic information

There were 85 participants including men and women community members, community and religious leaders, and health workers (Table 1). All the potential participants who were approached agreed to participate in the study. The sociodemographic characteristics of the participants is shown in Table 2.

**Table 2:** Age and sex distribution of the study participants.

**Sociodemographic characteristics**		**Frequency (*N* = 85)**
Sex	Men	47
	Women	38
Age (years)	18–25	15
	26–35	11
	36–45	29
	≥46	30
**Community members' distribution** **(*****n*** **=** **45)**		
Marital status	Single	22
	Married	23
Educational status	Less than basic education	15
	Basic education and higher	30
Occupation	Trading	17
	Artisans	13
	Students	12
	Unemployed	3

### Themes and subthemes

Four main themes were identified from the data as shown in Table 3. These themes are described in the following sections with supporting quotes.

**Table 3:** Themes derived from the data.

**Themes**	**Subthemes/description**
Why men's health requires attention	Participants' views on the need for specific attention being given to men's health. There were three subthemes:
	1. Preventing transmission of infections
	2. Providing support to partners
	3. Potential impact on the health of future children
The importance of addressing male infertility	Participants' opinions about the importance of specific health care for men. There were two subthemes:
	1. Health care for men should address infertility
	2. PCC for men can address potential causes of infertility
How men receive or respond to health care	Description of the ways in which men present to health facilities for health care and their response to the service provided
Opinions about men's need for PCC	Participants' opinions about the need for PCC among men. There were three subthemes identified:
	1. Men need PCC
	2. Men need PCC, but women need it more
	3. Men do not need PCC

#### The importance of male fertility

All the groups of participants believed that health care for men should include a focus on male infertility. The participants also stated that PCC may be an opportunity to address male infertility as highlighted in the two subthemes.

##### Health care for men should address infertility

At the community level, the concern for men's health was mainly about their role in the fertility equation with several participants describing the possibility of low sperm count and the need to address it. The health workers also spoke extensively about the need to address infertility in men because when couples have challenges with conception the usual impression is that the woman has a problem whereas the problem may be solved by addressing the man's health.

*Also, to buttress what they've been saying, men have to go for check-up too because there are some men that have low sperm count. I also think men should avoid certain food items that could cause low sperm count or that could hinder them from getting their wives pregnant.—* **FGD Participant1; Single Women Less than Basic Education FGD**

*Also, the sperm cells of the man may not be fertile. This can be treated if it is detected during tests by the doctor*.—**FGD Participant1; Married Women Basic Education and Above**

*It is also possible that the husband may have low sperm count and that may lead to infertility.—* **FGD Participant1; Single Men Less than Basic Education**

##### PCC for men can address potential causes of infertility

Following up oengn the need for men's health care to address infertility, some of the health workers held the view that PCC for men may be an opportunity to address potential causes of infertility. They however stated that most men are unaware of their need for such care and do not present to the health facility for it. They went further to state that there are no programs targeted at men's health specifically within the Nigerian health care space.

*Yes, they need it please. The reason is that some diseases occur in childhood that can cause infertility in men and most of the time they will say the problem is with the woman. So men too need to go to the hospital and check themselves, check their sperm counts*.—**Public Health Nurse; Primary Care Level**

*I believe men do though most of the time they do not present themselves or they are not aware they need such care too. I don't think they are really aware or maybe it's the Nigerian system. I don't think we have any program for men or young boys or any program to train them or prepare them for fatherhood*.—**Public Health Nurse; Tertiary Care Level (ICH)**

#### Why men's health requires attention

The participants gave their opinions about the importance of specific health care for men including prevention of transmission of infections to partners, providing support to partners, and the potential impact of men's health on future children.

##### Preventing transmission of infection

Some of the participants believed that there is a need for specific health services for men to screen and provide treatments that would prevent the transmission of infection to their partners.

*It is important for the man too to take care of himself before he sleeps with the woman and gets her pregnant and it is important that they go for check-up so that neither of them infects the other*.—**Female Community Leader**

##### Providing support to partners

The supportive role of men to their partners was highlighted as another reason for paying attention to men's health. During visits to health facilities, information on women's health could be given to the men, enabling them to provide evidence-based care to their partners.

*The youths, the husband and wife should both have preconception care in order to add to their knowledge so that they will know what to do per time. For example, they will be enlightened on the kind of food the wife must eat before and during pregnancy and the kind of work, she must do*.—**FGD Participant1; Single Men Basic Education and Above**

##### Potential impact on the health of future children

The pediatric specialists provided reasons for men requiring health care, mainly concerning the effect of men's health on the overall health of their families. The potential impact of a man's health on his contribution to the genetic pool of his child was stated. Also highlighted was the key role of men as breadwinners for their families which may be impacted negatively by poor health.

*Yes, the health of men of reproductive age is also important. Why is it important? This may sound a bit selfish but a man is typically the breadwinner in our setting. If he lives a short life that means the woman and the children are going to be in trouble. So, he has to be healthy so that he can take care of his wife financially and be the father figure for the children so they can be psychologically stable and secure*.—**Pediatric Cardiologist; Tertiary Care Level**

*Well, for example, psychological or mental health problems can affect the baby because the way the father views things and the decisions he takes can have negative or positive impact on the baby. Also, he will pass on some genes that will affect the baby*.—**Pediatrician; Tertiary Care Level**

#### How men receive or respond to health care

The health workers described men as being reluctant to seek health care. They stated that in their experience men tend to be less open about their reproductive health needs until a problem arises that they are unable to handle. Adolescent men often request help from health workers when pubertal changes begin and they need reassurance. Older men are often not seen in the clinics until they have questions about fertility which usually arise when their wives are unable to attain conception at the desired time.

…* Men will not come out the way women will just come out and say this is what I'm feeling. You know men, they will just be perambulating and until you send them for some tests, then they will come and start to ask questions after getting their results. That is when you can counsel on the issues; either low or zero sperm count*.—**Secondary Health Care Level Nurse**

*Mostly, you know men don't usually have problem and when they have, they don't own up on time but most of those we see are in the younger age group and that is where adolescent care comes in. During adolescence they start seeing changes in their bodies and are confused. Psychologically and emotionally you have to stabilize them and let them know that it's a normal phase. But for infertility in men, it usually comes up later in life and it is even the woman that presents first*.—**Family Physician; Tertiary Care Level**

#### Opinions about men's need for PCC

The opinions about whether men require PCC or not varied among the study population. While some participants believed men need PCC and should receive it, others believed that men do not need PCC at all or as much as women do.

##### Men need PCC

*Medical screening is necessary to prevent pregnancy complications*

At the community level, participants were of the opinion that PCC should not just be for women. Highlighting the man's role in pregnancy outcomes, they stated that the medical tests recommended during the preconception period should include both men and women. The possibility of low sperm counts leading to infertility came up in many of the discussions and was described as potentially amenable to PCC. The importance of the blood group and genotype in the occurrence of birth complications and having babies with sickle cell disorder was also mentioned.

*The couple must have some medical tests done. These include genotype test, blood group, and HIV test in order to prevent complications arising after marriage. It should not just be about women; men also need to be taken care of. In one of the programs that we just had, we found some men had health problems. Some had low sperm counts and did not know the cause until they visited the hospital. So, like has been said it is important for men to have medical check-ups too so that they will be in good health before pregnancy*.—**FGD Participant4; Christian Clergy**

*If the man has an infection, the new-born child will be affected by whatever disease he has. And if the man is healthy and the woman has a disease, their child will also be affected by that disease, because one of the sources from which the child is coming already has a defect. So, the man needs to examine himself, and go for a check-up to determine if he is healthy enough to be a father*.—**FGD Participant4; Muslim Clergy**

*From my perspective, it is important for both of them to know about preconception care when planning to get pregnant because both of them may belong to the blood group AS*.—**FGD Participant1; Single Men Less than Basic Education**

*The man that wants to impregnate his wife should go for a blood test. Some issues with their blood group and genotype can cause complications or they can give birth to a child with sickle cell. But if they have gone through a series of medical tests, the doctor will know the kind of treatment to be given to them after they have identified if they have any disease*.—**FGD Participant1; Married Women Basic Education and Above**

##### Men need PCC but women need it more

On the other hand, some the health workers expressed the opinion that although men require PCC, their reproductive health tends to be neglected in favor of women's health both socially and medically. This neglect of men's health in favor of women is believed to stem from the fact that women's health is believed to require more attention because they are the ones who become pregnant. From the cultural perspective, the expectations regarding fertility in society are often skewed more toward women than men.

*Men require care also but medically and also socially we tend to neglect the reproductive aspect of men's health unless they have problems with infertility and the like. However, I believe they also need preconception care*.—**Ob/Gyn; Secondary Care Level**

*Men also need preconception care but not as much as women*.—**Community Physician 1; Tertiary Care Level**

*It is usually when men have problems that we attend to them specially because even in our culture we don't believe they have problems with conception, it's usually women. You know, even if there is infertility between husband and wife, and it is the husband who has the problem, it is always assumed first that it is the woman*.— **Public Health Nurse; Tertiary Care Level (Community Medicine)**

##### Men do not need PCC

Some of the married men expressed the opinion that there is no need for men to have PCC. Men are expected to be healthy and care for their families and should not need special attention to their health, they opined.

*Well to my own understanding, we are the men of the house and if we are healthy, we should go out and look for what the family will eat. I have to go out because if I stay at home, there is nothing to eat. I don't think men need any special care*.—**FGD Participant1; Married Men Less than Basic Education**

Stating the assumption that men are generally healthy, some of the health workers were categorical in the expression of their belief that men do not need PCC. Some health workers stated that men would benefit from access to health care but not PCC while others were not aware of PCC services for men.

*Whether men need care that is different, I don't think they do except if there are special considerations*.—**Community Physician 2; Tertiary Care Level**

*They require care but not preconception care, just normal health care*.—**Pediatrician; Secondary Care Level**

*Interestingly, I am just hearing that for the first time. I have not heard about preconception care for men before. I didn't even know that services like that existed*.—**Ob/Gyn; Secondary Care Level [Private]**

*We see men of that age but we don't ask them if their wife is about to conceive; our aim for all patients really is to get optimal glucose control, blood pressure control, make sure they are optimally healthy at all times … maybe the urologists will be able to tell us better. But a normal man walking down the street I think they are healthy*.—**Endocrinologist; Tertiary Care Level**

## Discussion

This qualitative exploration revealed different views about men's need for PCC among the study participants. Identifying the importance of men's genetic contribution to pregnancy outcomes and men's role within the family, participants stated the belief that there is a need to pay attention to men's health. They however described men as being hesitant to seek health care, which poses a challenge to them receiving the attention they require timeously. Speaking specifically about PCC, opinions were diverse with respect to its relevance to men's health. While some of the participants believed that PCC is important for men, some stated that PCC is more essential for women than men while others disagreed emphasizing that men have no need for PCC.

Most African communities place a high premium on childbearing and this is the case in Nigeria where the study was done. Childbearing is important regardless of gender and pregnancy is culturally expected soon after marriage, although women are often the focus of attention in this regard more than men (30). The emphasis on low sperm counts and its impact on pregnancy desire is therefore logical in the context. Many of the participants viewed prevention and treatment of low sperm count and other potential causes of infertility as important reasons to address men's health. In addition, the importance of preventing transmission of infections to their partners and potentially transferring genetic diseases to their children was also highlighted. Research abounds on the genetic influences of men's environmental and lifestyle exposures and the impact of these on reproductive health outcomes (1, 7, 21–23). In societies where childbearing has a high value, PCC is relevant to addressing factors that can potentially cause delay or prevent the attainment of fatherhood.

Some of the participants also described the important roles men play in supporting their partners through pregnancy and childbirth once they have the needed information. PCC can be an opportunity to provide health information to couples ahead of pregnancy. Extant literature has documented that apart from being beneficial to the men themselves, providing PCC for men increases the likelihood of their female partners adhering to the lifestyle changes needed for optimal preconception health (10). For men who do not believe they need PCC, support for their partners' health can be a strategy to encourage them to access the service. Once in the system, they can then be provided with other services that will be beneficial for their health.

Similar to what is known for women, male fecundity and fertility can also be negatively impacted by risk factors such as stress, environmental toxins, excessive alcohol consumption, use of tobacco and other harmful substances, and obesity (7, 21–23). Unlike the services available for women through maternal health care, however, men's health has not been similarly addressed. Although there is some evidence that men who are preparing to be fathers may be concerned about their health, they may not be receiving adequate information about risk factors and critical issues because health care providers are not aware or trained to provide the service (10, 46). A study among Nigerian women with pre-existing medical conditions showed missed opportunities for PCC services despite regular contact with the health system on account of their past medical history (47). These factors, coupled with a lack of awareness among health care workers as described in this study may explain the reluctance with which men approach reproductive health services. To combat this reluctance, there is a need for improved awareness of the importance of men's overall and reproductive health among health care providers and at the community level.

Community and religious leaders are able to fill the gap in raising awareness about men's health at the community level. This is because besides exercising authority within their sphere of influence, the community members have implicit trust in them as sources of information and custodians of culture establishing and protecting traditional norms (48). Extant literature documents the importance role that community and religious leaders play as change agents and gatekeepers at the community level (49–51). Instances of this influence are largely related to maternal and child health, including family planning (49), prevention of HIV (48), protecting and improving child health, (51) among other health issues. An extension of their role to include men's health care is possible especially in communities where they provide premarital counseling. It is therefore important to involve community and religious leaders in the advocacy and awareness campaigns for men's health care including PCC.

The perception that PCC is more important for women than men has been documented previously where the participants stated that the term PCC appears to be targeted toward women because they are the ones who become pregnant (30, 52, 53). Participants in these studies opined that some health behaviors such as alcohol consumption and tobacco use were more relevant for men than women. They, therefore, believed PCC should be applicable directly to men and not only to women. In other studies, participants expressed the opinion that both men and women need to be involved in the pregnancy process right from the preconception period (17, 54). Similar perceptions could have influenced the different views expressed in the current study. Those who hold to the opinion that a more inclusive terminology is required are more likely to believe that there is a need for PCC services to be targeted at men compared with those whose opinion is that women require PCC in order to be prepared for pregnancy. Although PCC originally focused on women and the interventions provided addressed maternal health and access to health care, the benefits extended to neonatal, infant, and child health. The acknowledgment of the role of men's health has led to an expansion of PCC to include men's health (9). To improve uptake of PCC services among men, this understanding needs to be emphasized through training of health care providers and improved awareness within community settings.

This study is novel in its presentation of viewpoints about men's reproductive health needs with a focus on PCC. Although limited in terms of the context of the study, the perception among the health care providers, community members and community and religious leaders has not been documented previously. The viewpoints expressed may be therefore transferable to similar contexts within many LMICs. The also raises questions for further research into factors influencing men's healthcare seeking behavior which may be explored at community and health service level.

## Conclusion

Although many of this study's participants identified the need to provide health services for men, reluctance to access health services among men was highlighted. This reluctance could be due to a lack of perception of need among men, especially with regard to PCC. The views proffered concerning men's need for PCC varied from an agreement that PCC is required to denial of the need for such services among men. This variation in opinion cut across the different participant groups highlighting the need for improved awareness about men's reproductive health needs both among health workers and at the community level. This variation speaks to the low level of attention given to men's reproductive health. In addition, the lack of inclusion of men's health in health policies and programs may explain some of the opinions expressed in this study. Since PCC service provision is still rudimentary in Nigeria, successful deployment and uptake will require inclusion of men's need for PCC in health-related policy updates, adequate training of health workers on PCC services and improved awareness at the community level. Future research may examine the possible role of community and religious leaders in encouraging men's uptake of reproductive health care and the acceptability of PCC services for men on a broader scale across other communities in sub-Saharan Africa.

## Data availability statement

The original contributions presented in the study are included in the article, further inquiries can be directed to the corresponding author.

## Ethics statement

The studies involving human participants were reviewed and approved by Oyo State Ministry of Health (Approval number AD/13/479/565), the University of Ibadan/University College Hospital (UI/UCH) Institution Review Board (UI/EC/17/0390), and the Wits Human Research Ethics Committee (Medical; Clearance number M171054). The patients/participants provided their written informed consent to participate in this study.

## Author contributions

OO and LI: study concept and design, analysis and interpretation of data, critical revision of the manuscript for important intellectual content, and final approval of the manuscript. OO: acquisition of data and drafting of the manuscript.

## Conflict of interest

The authors declare that the research was conducted in the absence of any commercial or financial relationships that could be construed as a potential conflict of interest.

## Publisher's note

All claims expressed in this article are solely those of the authors and do not necessarily represent those of their affiliated organizations, or those of the publisher, the editors and the reviewers. Any product that may be evaluated in this article, or claim that may be made by its manufacturer, is not guaranteed or endorsed by the publisher.

## References

[1] Toivonen KI Oinonen KA Duchene KM. Preconception health behaviours: a scoping review. Prev Med. (2017) 96:1–15. 10.1016/j.ypmed.2016.11.022 27939264

[2] Verbiest S Shawe J Steegers EAP. Advancing preconception health globally: a way forward. In:J Shawe, editor, Preconception Health and Care: A Life Course Approach. Cham: Springer Nature Switzerland AG (2020). p. 299–308. 10.1007/978-3-030-31753-9_15

[3] World Health Organization. Meeting to Develop a Global Consensus on Preconception Care to Reduce Maternal and Childhood Mortality and Morbidity. WHO Headquarters, Geneva Meeting Report. Geneva: World Health Organization (2012).

[4] Omigbodun AO. Preconception care in Nigeria: prospects and constraints. Archiv Ibadan Med Bookbuild. (2002) 3:3–5. 10.4314/aim.v3i1.34568

[5] Frey KA Navarro SM Kotelchuck M Lu MC. The clinical content of preconception care: preconception care for men. Am J Obstet Gynecol. (2008) 199:S389–95. 10.1016/j.ajog.2008.10.024 19081435

[6] Buck Louis GM Barr DB Kannan K Chen Z Kim S Sundaram R. Paternal exposures to environmental chemicals and time-to-pregnancy: overview of results from the LIFE study. Andrology. (2016) 4:639–47. 10.1111/andr.12171 27061873 PMC4961554

[7] Kothari A Thayalan K Dulhunty J Callaway L. The forgotten father in obstetric medicine. Obstet Med. (2019) 12:57–65. 10.1177/1753495X18823479 31217809 PMC6560841

[8] Atrash H Jack BW Johnson K. Preconception care: a 2008 update. Curr Opin Obstet Gynecol. (2008) 20:581–9. 10.1097/GCO.0b013e328317a27c 18989135

[9] Garfield CF. Toward better understanding of how fathers contribute to their offspring's health. Pediatrics. (2018) 141:e20173461. 10.1542/peds.2017-3461 29229681

[10] O'Brien AP Hurley J Linsley P McNeil KA Fletcher R Aitken JR. Men's preconception health: a primary health-care viewpoint. Am J Mens Health. (2018) 12:1575–81. 10.1177/1557988318776513 29774805 PMC6142132

[11] Choiriyyah I Sonenstein FL Astone NM Pleck JH Dariotis JK Marcell AV. Men aged 15–44 in need of preconception care. Matern Child Health J. (2015) 19:2358–65. 10.1007/s10995-015-1753-7 26112749

[12] Abrha MW Asresu TT Weldearegay HG. Husband support rises women's awareness of preconception care in northern Ethiopia. Scientific World J. (2020) 2020:1–7. 10.1155/2020/3415795

[13] Casey FE Sonenstein FL Astone NM Pleck JH Dariotis JK Marcell AV. Family planning and preconception health among men in their mid-30s developing indicators and describing need. Am J Mens Health. (2016) 10:59–67. 10.1177/1557988314556670 25389215 PMC4490119

[14] Dean S V Lassi ZS Imam AM Bhutta ZA. Preconception care: promoting reproductive planning. Reprod Health. (2014) 11:S2. 10.1186/1742-4755-11-S3-S2 25415259 PMC4196558

[15] Coffey K Shorten A. The challenge of preconception counseling: using reproductive life planning in primary care. J Am Assoc Nurse Pract. (2014) 26:255–62. 10.1002/2327-6924.12054 24170712

[16] Grace B Shawe J Johnson S Stephenson J. You did not turn up… I did not realise I was invited…: understanding male attitudes towards engagement in fertility and reproductive health discussions. Hum Reprod Open. (2019) 2019:1–7. 10.1093/hropen/hoz014 31218265 PMC6573469

[17] Poels M Koster MPH Franx A van Stel HF. Parental perspectives on the awareness and delivery of preconception care. BMC Pregnancy Childbirth. (2017) 17:9–11. 10.1186/s12884-017-1531-1 28950838 PMC5615801

[18] Hamm M Evans M Miller E Browne M Bell D Borrero S. “It's her body”: low-income men's perceptions of limited reproductive agency. Contraception. (2019) 99:111–7. 10.1016/j.contraception.2018.10.005 30336131 PMC6744607

[19] Hammarberg K Collins V Holden C Young K McLachlan R. Men's knowledge, attitudes and behaviours relating to fertility. Hum Reprod Update. (2017) 23:458–80. 10.1093/humupd/dmx005 28333354

[20] Martín-Calvo N Mínguez-Alarcón L Gaskins AJ Nassan FL Williams PL Souter I. Paternal preconception folate intake in relation to gestational age at delivery and birthweight of newborns conceived through assisted reproduction. Reprod Biomed. (2019) 39:835–43. 10.1016/j.rbmo.2019.07.005 31564651 PMC6848767

[21] Segal TR Giudice LC. Before the beginning: environmental exposures and reproductive and obstetrical outcomes. Fertil Steril. (2019) 112:613–21. 10.1016/j.fertnstert.2019.08.001 31561863

[22] Braun JM Messerlian C Hauser R. Fathers matter: why it's time to consider the impact of paternal environmental exposures on children's health. Curr Epidemiol Rep. (2017) 4:46–55. 10.1007/s40471-017-0098-8 28848695 PMC5571868

[23] Kotelchuck M Lu M. Father's role in preconception health. Matern Child Health J. (2017) 21:2025–39. 10.1007/s10995-017-2370-4 28983715

[24] Stephenson J Fleming TP Yajnik CS Prentice AM Forrester T Barker M. Origins of lifetime health around the time of conception: causes and consequences. Lancet. (2018) 391:1842–52. 10.1016/S0140-6736(18)30312-X 29673874 PMC5975952

[25] Stephenson J Patel D Barrett G Howden B Copas A Ojukwu O. How do women prepare for pregnancy? Preconception experiences of women attending antenatal services and views of health professionals. PLoS ONE. (2014) 9:103085. 10.1371/journal.pone.0103085 25058333 PMC4109981

[26] National Population Commission ICF. Nigeria Demographic and Health Survey 2018. Abuja; Rockville, MD (2019).

[27] Sekoni OO Ojifinni OO Adediran OS. Spousal communication and family planning use among married adults in a rural community in Nigeria. Nigerian J Public Health. (2017) 2:139–50.

[28] Smith DJ. Masculinity, money, and the postponement of parenthood in Nigeria. Popul Dev Rev. (2020) 46:101–20. 10.1111/padr.12310 34290463 PMC8290976

[29] Ntoimo LFC Isiugo-Abanihe U. Patriarchy and singlehood among women in Lagos, Nigeria. J Fam Issues. (2014) 35:1980–2008. 10.1177/0192513X13511249

[30] Ojifinni OO Ibisomi L. Preconception care practices in Nigeria: a descriptive qualitative study. Reprod Health. (2020) 17:172. 10.1186/s12978-020-01030-6 33148313 PMC7640668

[31] Adesina K Aderibigbe S Fawole A Ijaiya M Olarinoye A. Pregnancy outcome of the obese in Ilorin. Obstet Med. (2011) 4:160–3. 10.1258/om.2011.100081 27579116 PMC4989639

[32] Creswell JW Poth CN. Qualitative Inquiry and Research Design: Choosing Among Five Approaches. 4th ed. Thousand Oaks, CA: Sage Publications Inc. (2018).

[33] Green J Thorogood N. Qualitative methods for health research. 3rd ed. In: J Seaman, L Mehrbod, I Antcliff, K Harrison, editors, Thousand Oaks, CA: Sage Publications, Inc. (2014).

[34] Heale R Forbes D. Understanding triangulation in research. Evid Based Nurs. (2013) 16:98–98. 10.1136/eb-2013-101494 23943076

[35] Federal Ministry of Health Nigeria. National Health Policy 2016: Promoting the Health of Nigerians to Accelerate Socioeconomic Development. Abuja (2016).

[36] National Primary Health Care Development Agency (NPHCDA). Minimum Standards for Primary Health Care in Nigeria. Abuja (2012).

[37] Aigbiremolen AO Alenoghena Eboreime E Abejegah C. Primary health care in Nigeria: from conceptualization to implementation. J Medical Appl Biosci. (2014) 6:35–43.35777803

[38] Wekesah F Izugbara C. Maternal Health in Nigeria: Facts Figures. African Population Health Research Centre Fact Sheet. (2017). Available online at: http://aphrc.org/wp-content/uploads/2017/06/APHRC-2017-fact-sheet-Maternal-Health-in-Nigeria-Facts-and-Figures.pdf

[39] Jayasekara RS. Focus groups in nursing research: methodological perspectives. Nurs Outlook. (2012) 60:411–6. 10.1016/j.outlook.2012.02.001 22464693

[40] Winke P. Using focus groups to investigate study abroad theories and practice. System. (2017) 71:73–83. 10.1016/j.system.2017.09.018

[41] Tong A Sainsbury P Craig J. Consolidated criteria for reporting qualitative research: a 32-item checklist for interviews and focus groups. Int J Qual Health Care. (2018) 19:349–57. 10.1093/intqhc/mzm042 17872937

[42] Goldblatt H Karnieli-Miller O Neumann M. Sharing qualitative research findings with participants: study experiences of methodological and ethical dilemmas. Patient Educ Couns. (2011) 82:389–95. 10.1016/j.pec.2010.12.016 21257280

[43] Fereday J Muir-Cochrane E. Demonstrating rigor using thematic analysis: a hybrid approach of inductive and deductive coding and theme development. Int J Qual Methods. (2006) 5:80–92. 10.1177/160940690600500107

[44] Braun V Clarke V. Using thematic analysis in psychology. Qual Res Psychol. (2006) 3:77–101. 10.1191/1478088706qp063oa

[45] VERBI Software. MAXQDA. 2018. Berlin: VERBI Software (2017).

[46] Frey KA Engle R Noble B. Preconception healthcare: what do men know and believe? J Mens Health. (2012) 9:25–35. 10.1016/j.jomh.2011.11.00133228762

[47] Ojifinni OO Ibisomi L. Exploring the need for preconception care: the pregnancy experiences of women with pre-existing medical conditions in Ibadan, Nigeria. Afr J Reprod Health. (2021) 25:28–38. 10.21203/rs.3.rs-18571/v1 37585751

[48] Chimatiro CS Hajison P Muula AS. The role of community leaders on adolescent's HIV and sexual reproductive health and rights in Mulanje, Malawi. Reprod Health. (2020) 17:8. 10.1186/s12978-020-00917-8 32408906 PMC7226947

[49] Adedini SA Babalola S Ibeawuchi C Omotoso O Akiode A Odeku M. Role of religious leaders in promoting contraceptive use in Nigeria: evidence from the Nigerian urban reproductive health initiative. Glob Health Sci Pract. (2018) 6:500. 10.9745/GHSP-D-18-00135 30287529 PMC6172128

[50] Fantaye AW Okonofua F Ntoimo L Yaya S. A qualitative study of community elders' perceptions about the underutilization of formal maternal care and maternal death in rural Nigeria. Reprod Health. (2019) 16:5. 10.1186/s12978-019-0831-5 31711527 PMC6849176

[51] Eyber C Kachale B Shields T Ager A. The role and experience of local faith leaders in promoting child protection: a case study from Malawi. Intervention. (2018) 16:31. 10.1097/WTF.0000000000000156

[52] Ojifinni OO Munyewende PO Ibisomi L. Exploring the perception of and attitude towards preconception care service provision and utilisation in a South Western Nigerian community – a qualitative study. Afri Popul Stud. (2021) 35:S102. 10.11564/35-1-1529

[53] Tuomainen H Cross-Bardell L Bhoday M Qureshi N Kai J. Opportunities and challenges for enhancing preconception health in primary care: qualitative study with women from ethnically diverse communities. BMJ Open. (2013) 3:1–9. 10.1136/bmjopen-2013-002977 23883884 PMC3731794

[54] Bayrami R Latifnejad Roudsari R Allahverdipour H Javadnoori M Esmaily H. Experiences of women regarding gaps in preconception care services in the Iranian reproductive health care system: a qualitative study. Electron Physician. (2016) 8:3279–88. 10.19082/3279 28070262 PMC5217821

